# Social accountability challenges and recommendations by community service rehabilitation therapists

**DOI:** 10.4102/safp.v66i1.5936

**Published:** 2024-06-06

**Authors:** Ntandoyenkosi L. Msomi, Andrew J. Ross

**Affiliations:** 1 Department of Family Medicine, College of Health Sciences, University of KwaZulu-Natal, Durban, South Africa

**Keywords:** social accountability, community service rehabilitation therapists, challenges, recommendations, rehabilitation services

## Abstract

**Background:**

Social accountability entails providing equitable and accessible services that are tailor-made for the community’s healthcare needs and enable rehabilitation therapists to improve the efficiency and efficacy of healthcare delivery and their response. Enabling them to provide optimal care during their community service year requires understanding the gaps in their knowledge, experience and the support they provide to the communities they service.

**Methods:**

Four in-depth individual interviews and four focus group discussions were conducted via Zoom. The qualitative responses to the questions related to the challenges and recommendations associated with social accountability in clinical settings were analysed using an inductive thematic approach via NVIVO.

**Results:**

Four sub-themes emerged for each of the two areas of interest: the challenges relating to (1) budget and equipment constraints, (2) staff shortages, (3) cultural and language barriers and (4) scope of practice limitations. The recommendations related to (5) collaboration with community caregivers, (6) service inclusion in primary health care clinics, (7) improved executive management support and (8) continuing professional development.

**Conclusion:**

Equipping graduates with the knowledge, skills and support needed to work in an under-resourced setting is essential for community service rehabilitation therapists to ensure social accountability, given that they often work alone, specifically in rural settings.

**Contribution:**

Being aware of the challenges that face community service rehabilitation therapists, having the necessary tools and health facility management support will enable ongoing improvements in their ability to provide socially accountable services.

## Introduction

South African rehabilitation graduates must complete a compulsory year of community service before being able to register as independent practitioners with the local regulatory body, the Health Professions Council of South Africa (HPCSA). The National Department of Health implemented the community service year in an effort to deliver healthcare services to resource-constrained communities in rural and peri-urban areas in particular. Rehabilitation professions consist of those health disciplines that focus on the modification of impairment, compensation for loss of function and modification of the environment: these are audiology, occupational therapy, physiotherapy and speech-language therapy.^1^ Community service rehabilitation therapists consult with patients in hospitals and primary health care clinics, and engage in community-based rehabilitation, which can take the form of home consultations, health promotional talks in community halls and healthcare screening programmes in schools. The therapists are exposed to community-based rehabilitation during their undergraduate training through community-based education initiatives, which aim to provide opportunities for self-development and enhanced clinical and problem-solving skills and stimulate self-directed lifelong learning.^2^ During such activities, the student therapists are generally supervised by senior therapists in their disciplines or from a different cadre when there are staff shortages.

While knowledge is regarded as a state of understanding a specific situation, place or experience, practice is enacting a belief or acquired knowledge. Knowledge and practice are therefore complementary and central to fulfilling health science graduates’ competencies of being (1) an effective communicator, (2) a lifelong scholar, (3) a patient-centred collaborator, (4) an active health advocate and (5) a prudent leader and manager.^3^ The World Health Organization (WHO) regards the knowledge and practices of healthcare professionals as essential for them to advocate for people’s right to healthcare, without any form of gender, religious or ethnic-based discrimination.^4^ The South African constitution embedded a similar ethos by including people’s right to access healthcare.^5^

When systemic and public health inefficiencies, such as inadequate planning and poor service delivery, are encountered, social accountability can provide a framework to manage them. Social accountability is defined by Malena et al. as ‘an approach towards building accountability that relies on civic engagement, i.e. in which it is ordinary citizens and/or civil society organizations that participate directly or indirectly in exacting accountability’. While there are many and varied mechanisms to enable such accountability, it requires access to relevant data that can be analysed and disseminated to mobilise public support that will affect desired changes.^6^ In order for graduates to understand the implications of this approach for their year of community service, instruction at the undergraduate level needs to be supplemented by training and experience. Boelen et al.^7^ contend that for their graduates to enable social accountability, it is:

[*T*]he obligation of the faculty to direct their education, research, and service activities towards addressing the priority health concerns of the community, the region, or the nation they have the mandate to serve. (p. 3)

This means that universities and relevant stakeholders must ensure that (1) training in health sciences is contextualised locally and they graduate fit-for-purpose healthcare professionals, (2) community needs analysis is jointly conducted by healthcare professionals, service provision organisations and community members and (3) there are regular public health service delivery reviews. Community service rehabilitation therapists need to gain competency in communication skills, cultural appreciation, asset inventory compilation, stakeholder collaboration and health needs profiling to enable them to conduct community needs assessments.^2^ While the sentiments of social accountability should be commonly understood and practised, most public sector departments limit their activities to social mobilisations and audits, participatory budgeting and information-sharing campaigns.^8^ The World Bank Development offers a social accountability framework that consists of three key elements: (1) the state, which includes politicians and policymakers, (2) healthcare providers, consisting of frontline workers and organisations and (3) citizens who are classified as poor and non-poor. The interaction of providers and citizens ensures responsive service delivery, while the interaction of citizens and the state ensures the discussions of community concerns.^9^

Various factors contribute to healthcare professionals’ understanding of social accountability, such as training, use of technological advances in clinical practice, understanding patients’ rights, personal beliefs, core values, realising social health inequalities^10,11,12^ and equitably delivering services equivalent to the degree of need. Internationally, healthcare professionals practice social accountability in multiple ways, such as engaging with immediate health-related societal needs, reviewing the efficiency of services and devising health service delivery plans. An emphasis on improving health sciences education’s response to societal health needs was central to the global consensus for social accountability instruction at medical schools.^13^

The recent COVID-19 pandemic intensified social media engagement among healthcare professionals, reflecting their active involvement in disseminating crucial health information and addressing pressing public concerns.^14,15^ Healthcare professionals in community settings have used health promotional talks, and adapted communication techniques and cross-disciplinary role release, to identify and manage pressing societal health issues.^16,17^ Cross-disciplinary role release entails information sharing and basic clinical skill coaching outside a specific discipline, this being relevant where there are staff shortages, such as in rural areas, and facilitates improved management of community healthcare needs.

There is limited information on the social accountability practices among rehabilitation therapists working in KwaZulu-Natal (KZN) province, South Africa, the province being the second most populous after Gauteng, with an estimated 54% of its 12.4 million people living in rural areas.^18^ The majority of the KZN population (79%) access health care through the public sector,^19^ most of which are under-resourced and understaffed, making it difficult for them to timeously meet the needs of the people they serve.^20^ In rural areas in particular, access to healthcare facilities for people with disabilities is also impacted by unreliable public transport and long queues.^21^ Primary health care services are available in rural areas, with referrals being required to district-level hospitals at which community service rehabilitation therapists are based, of which there are 23 in KZN, with upward referral to more specialised levels of care. Some rehabilitation therapists visit clinics or undertake community projects on a rotational or appointment basis, for example once a month, with access to such services being constrained for those who cannot afford to travel. Some rehabilitation therapists identify community initiatives, such as Community Based Rehabilitation (CBR) initiatives, which they feel are important to pursue as part of their work obligations.

Newly graduated rehabilitation therapists are often the only people in their discipline providing services at district-level hospitals, being required to learn not only how to run a department, but to work without the support of senior staff and advisors. In addition to executing their normal daily activities, implementing social accountability initiatives in communities whose culture and language they are not familiar with can pose a challenge.

In this regard, there is little understanding of what challenges are encountered by rehabilitation therapists during their community service year while implementing social accountability interventions in resource-constrained communities. This study therefore aimed to explore the challenges the community service rehabilitation therapist faced in implementing social accountability while working in the public health sector in KZN, and their recommendations to overcome them.

## Research methods and design

A qualitative, interpretive and exploratory design was employed using focus group discussions (FGDs) and in-depth interviews (IDIs) to gain insight into the social accountability practices of community service rehabilitation therapists in KZN. The study was conducted in the iLembe, eThekwini, Harry Gwala, King Cetswayo, Ugu and Zululand Districts where community service rehabilitation therapists were located during their community service year.^22^ The KZN Department of Health provided a list of relevant rehabilitation therapists working in the six districts, with 30 being identified as potential participants. The inclusion criteria were that they needed to be registered with the regulatory body, the HPCSA, as community service audiologists, speech-language therapists, occupational therapists and physiotherapists.

Permission was obtained to present a poster invitation for the study on the encrypted WhatsApp and Facebook groups for community service rehabilitation therapists. The potential participants who responded were emailed the information study sheet and consent form. Emails were also sent to the audiology, speech-language therapy, occupational therapy and physiotherapy departments in the identified six districts with study information sheets and consent forms. Of the 30 who were invited, 27 indicated a willingness to participate across the six districts, submitted signed consent forms via email and indicated which they preferred to participate in, an focus group participant (FGP) or semi-structured individual in-depth interviews (IDI).

Four FGDs were held virtually, because of a number of community service rehabilitation therapists being based at different facilities, and the four IDIs being held where only one therapist from a facility responded. Both were conducted remotely in English, with an option to respond in isiZulu, via Zoom’s virtual platform from July 2023 to October 2023, with the same interview guide used for both to ensure that participants were asked the same questions. The questions related to their understanding of social accountability, the challenges related to its implementation and recommendations on how to address them. The four FGDs and four IDIs were recorded with the participants’ permission, and an inductive thematic analysis was used to analyse the verbatim transcriptions and the different data sources enabling data triangulation to strengthen the validity of the results.^23^ The in-depth IDS employed a free attitude interviewing technique to obtain rich data by allowing participants to freely discuss anything related to the topics.^24^

Two researchers analysed the qualitative data separately using NVIVO (QSR International, Victoria, Doncaster, Australia) by repeatedly reading the transcripts to become familiar with the content. Thereafter, the transcripts were coded separately, these being combined to form emerging categories and themes. The involvement of an independent qualitative coder increased data trustworthiness and reduced bias, as the researcher at the time of study conceptualisation was also a community service speech-language therapist working in the eThekwini District in KZN.^25^ Discrepancies in codes were discussed until a collective decision was reached, the similarity of themes indicating that data saturation had been reached.

### Ethical considerations

Ethical approval to conduct the study was obtained from the Humanities and Social Sciences Research Ethics Committee of the University of KwaZulu-Natal (reference no.: HSSREC/00005538/2023). Gatekeeper permission was granted by the KZN Department of Health’s Deputy Director of Disability and Rehabilitation Services (HSSREC/00005538/2023) and Research Committee (reference no.: KZ_202305_030). All participants submitted written informed consent.

## Results

Their demographic details are presented in Table 1, which indicates that all 27 participants were South African-trained, mainly female (*n* = 22, 81%) and aged 22–30 years. There were slightly more speech-language therapists (*n* = 9, 34%), 59% were located in rural areas, with most (*n* = 21, 78%) having been trained at the University of KwaZulu-Natal.

**TABLE 1:** Participants demographics (*N* = 27).

Variable	Characteristic	*n*	%
First language	IsiZulu	19	70
	Other languages	8	30
Gender	Female	22	81
	Male	5	19
Ages	22–26 years	25	93
	27–30 years	2	7
Professions	Audiology	6	22
	Occupational therapy	6	22
	Speech-language therapy	9	34
	Physiotherapy	6	22
Location	Rural	16	59
	Peri-urban	11	41
Training institutions	University of Cape Town	3	11
	University of KwaZulu-Natal	21	78
	University of Pretoria	3	11

Table 2 shows the themes and associated sub-themes related to the challenges and recommendations to implement social accountability. To ensure anonymity, each participant was allocated a unique code, with those in FGDs being indicated as FGP and in the IDI.

**TABLE 2:** Summary of themes and sub-themes.

Themes	Sub-themes
1. Challenges relating to social accountability	1.1 Budget and equipment constraints 1.2 Staff shortages 1.3 Cultural and language barriers 1.4 Scope of practice limitations
2. Recommendations to implement social accountability	2.1 Collaborate with community caregivers 2.2 Service inclusion in primary health care clinics 2.3 Executive management support 2.4 Continuing professional development

### Theme 1: Challenges relating to social accountability

Four sub-themes emerged from the interviews and group discussions: rehabilitation budget constraints, staff shortages, cultural and language barriers, and scope of practice limitations.

#### Sub-theme 1.1: Budget and equipment constraints

Participants described the challenge imposed by annual budget allocations for rehabilitation services as including the lack of transport to provide them where they are needed and assistive device order cancellations. They also noticed that the lack of diagnostic tools for patient management made it difficult to objectively treat those affected:

‘… where I work, they tell you there are budget constraints regarding no vehicles to use for CBR [*community based rehabilitation*]. So, it is difficult to apply things, like making our services more accessible to people via home visits. They also do not accept donations because it will make the provincial department of health decrease the annual budgets.’ (IDIP2)

‘… we ordered wheelchairs and buggies last year. It was financially approved, and then someone canceled the order for some unknown reason, leaving patients from the majority of 2022 without any wheelchairs or buggies, and now it is 2023.’ (IDIP4)

#### Sub-theme 1.2: Staff shortage

The participants perceived the lack of sufficient staff as being a challenge to provide the desired level of services and achieve social accountability. Most narrated that many institutions rely solely on community service graduate rehabilitation therapists to staff their departments, this not being ideal because of their lack of experience and only having a 1-year contract, with no long-term, experienced permanent staff providing supervision in many facilities. The sentiments below were shared:

‘I was the only OT. I did not have a senior or supervisor as I was straight from school. Many needed OT services, but I was the only staff member.’ (FG1P1)

‘Three years ago, my hospital had a permanent speech therapist, but after that person resigned, they just use us community service therapists and we have motivated for posts multiple times with no success from the medical manager.’ (FG4P2)

#### Sub-theme 1.3: Cultural and language barrier

The participants indicated that the patients used their cultural practices and religious beliefs as a reference to how they perceived the health information given by graduate rehabilitation therapists, which had implications for how they follow-up with treatment recommendations. Two participants referenced scenarios by saying:

‘I had a patient who I suspected had ASD [*autism spectrum disorder*] and needed to be referred, but the family thought it was rather bewitchment or an ancestral calling.’ (FG3P4)

‘For me also, I have noticed that most people have their own beliefs. So, most of them believed that some of these disorders that we deal with were punishment from the ancestors.’ (FG3P2)

Other participants highlighted language differences as a challenge, and that in the absence of being able to communicate effectively, they were concerned about how the information was being received and acted upon:

‘I do not speak isiZulu. I found it very difficult, and of course, I cannot expect my patients to understand English. When I go into more rural places, it becomes even more difficult because they do not even hear it at all, whereas it is in more of, like I would not want to say, the more urban areas they hear it.’ (IDIP4)

#### Sub-theme 1.4: Scope of practice limitations

The participants described the challenges with their profession-specific scopes of practice, with most feeling that CBR required them to offer services beyond their scope limits. They specifically referred to medication, such as antibiotics and anti-inflammatories, which were needed by patients they saw with problems such as ear infections or joint problems, respectively, but could not prescribe for. This resulted in the patients having to go to their local clinic to access the medication, which required another trip and more waiting in queues, first to be seen by a health worker and then to access the medication. The participants shared that:

‘I see a patient with an ear infection, and I refer the patient to the hospital or clinic for a doctor because I cannot prescribe antibiotics and some other medications.’ (FG4P1)

‘Sometimes patients need anti-inflammatory meds, but I cannot assist them, so they still have to go to the hospital or the nearest clinic.’ (FG1P4)

### Theme 2: Recommendations to implement social accountability

In addition to the challenges indicated above, the participants shared strategies they felt could be implemented to ensure social accountability, these being collaboration with community caregivers, service inclusion in primary health care clinics, executive management support and continuing professional education.

#### Sub-theme 2.1: Collaboration with community caregivers

According to participants, because of the difficulties they had experienced with providing services to patients who needed rehabilitation services in the communities, working collaboratively with community caregivers could enable them to develop and implement plans to strategically meet the social accountability demands. Working in isolation of relevant community members had resulted in their services not reaching those who needed them the most. A participant said:

‘Community caregivers frequently see the patients and can assist with identifying those in need of rehab services.’ (IDIP1)

#### Sub-theme 2.2: Service inclusion in primary health care clinics

Current CBR services are provided at primary health care clinics once a month but only over 4 h, and this amount of time is inadequate to meet the needs of those who need to be seen, either for one appointment or on a regular basis. While appointments were made for patients each month, there were often too many to be seen by one person, making it necessary to reconsider where and how social accountability was addressed. The participants recommended the provision of these services at primary health care clinics on a regular basis, such as twice a month or for longer hours, to enable patients with disabilities to have easy access to their services, with visits to the nearest hospital being required if equipment was needed that was not available at the clinic. The participants observed:

‘CBR needs to be within the community and always available to people; PHCs need rehabilitation facilities. This will help us to be socially accountable.’ (IDIP2)

‘You need more than one therapist per discipline to work efficiently; one can remain at the hospital while the other visits clinics and communities.’ (FG2P4)

#### Sub-theme 2.3: Executive management support

Senior management appeared to lack an understanding of the benefits of rehabilitation services, as well as how, where and when they need to be provided to those in need, and who often need assistance to access them. The participants felt that an understanding of rehabilitation services by senior managers in health institutions was essential to implement changes and be open to recommendations. Participants felt that management’s focus on medical solutions, and the resulting lack of consultation and support for rehabilitation services, was a challenge that needed to be addressed:

‘Medical managers do not know what we do; how is it that only rehab does not have resources?’ (FG3P6)

‘A rehabilitation therapist should be in charge of a rehabilitation department, not a medical doctor; this will prevent communication breakdowns. We have to explain why a child with Cerebral Palsy needs a buggy.’ (FG4P6)

‘I am certain that cash flow meetings do not consider the rehabilitation department; only nursing and the medical component are important.’ (FG2P6)

#### Sub-theme 2.4: Continuing professional development

The participants felt that while their undergraduate training had provided them with the basic knowledge needed to perform their tasks as rehabilitation therapists, they were not fully prepared to work in the public sector environment. As their clinic experience during their training had been at urban hospitals, supervised by senior staff, they were not prepared for the challenges they would experience when working in rural facilities on their own. Given the large number of graduates who were working in such environments, they indicated the need to undertake training through formal opportunities, such as courses as part of their continuing professional development (CPD) education. They felt that some issues were common to rehabilitation professionals, such as understanding health and healing in a multicultural context and social services (disability grant payments), while others would be subject-specific.

Having to address problems without the full set of tools to provide optimal services prevented them from being able to manage their patients effectively, such as providing anti-inflammatory tablets or antibiotics. Being able to offer the same level of services as sisters at PHC in terms of medication dispensing, with limitations for their needs, would improve their ability to manage patients in situations where access was a challenge for both the patients and the therapists. They wanted this to be part of their undergraduate training and ongoing CPD, knowing that they would be spending a year in community service in resource-constrained settings. They also wanted the hospitals at which they were placed to provide more comprehensive induction programmes to prepare them for each environment, and how best to achieve social accountability:

‘The community needs are so complex that you have to be acquainted with a bit of everything; I mean basic pharmacology, neurodevelopment, social services, transport, the list is endless.’ (FG3P1)

‘They need more information apart from the disorders we are treating; home visits can be influenced by SASSA payout dates and spam messages shared on WhatsApp, so you must be knowledgeable about most stuff.’ (FG1P6)

## Discussion

The participants in this study regarded the rehabilitation department’s budget constraints as a challenge that restricted their opportunities for being socially accountable. The South African public healthcare facility managers allocate budgets to various institutional components through annual cash flow meetings, which also approve resource procurement requests. The participants highlighted that budget constraints were not merely institutional but systematic, as national budgets for health have decreased in real terms over the last decade.^26^ Resource constraints for healthcare services, particularly rehabilitation services, is a common concern globally, and not limited to the South African context.^27^ This has led to high ‘out-of-pocket’ patient payments to procure assistive devices that should routinely be provided but were not because of budget constraints, such as crutches and hearing aids.^28^ A study conducted in West Asia in 2015 described how the financial hardships experienced by rehabilitation departments disrupted the continuation of services and contributed to a national increase in disability rates. In this study, participants regarded financial and resource constraints as impeding their ability to be socially accountable to people in need of rehabilitation services.^29^ A study in Iran showed that rehabilitation services were not allocated sufficient financial resources,^28^ the findings being similar to this study, which impacted their ability to provide rehabilitation services in rural and peri-urban KZN communities.

The participants felt that staff shortages negatively impacted their ability to provide socially accountable services and the reliance on community service rehabilitation therapists with a 1-year contract led to a lack of continuity of services. In addition, the lack of senior rehabilitation professionals to supervise and mentor the clinical growth of community service therapists resulted in each new intake having to learn the same lessons every year. A study conducted across four countries (Bangladesh, Portugal, Singapore and the United States) highlighted that this is not unique to South Africa, with rehabilitation therapists often being concentrated in urban areas, resulting in rural communities being under-serviced.^30^ Consistent with this study, Gupta and colleagues further described that the number of rehabilitation therapists in low- and middle-income countries was inadequate, most of these countries being in sub-Saharan Africa.^31^ The study described that the lack of senior staff and poor support from the hospital management often resulted in rehabilitation services not providing input into budget requirements, which had the effect of their not being regarded as important as other medical services.

Some of the participants who were not isiZulu first language speakers felt that the language and cultural barriers impacted their ability to meet the rehabilitation needs of the local communities they served. The dominant language in KZN is isiZulu, and Zulu people in rural settings often only speak their mother tongue, with their health beliefs and practices being deeply rooted in their cultural norms. Participants reported a low uptake of effective therapy services if patients, or their families, felt that their illness was because of spiritual or ancestral issues. Translators were not provided by the hospitals, which resulted in frustration and communication challenges for both the community members and therapists. A study by Messias et al. found that language barriers greatly increased health disparities, contributed to uneven access to healthcare, poor health outcomes and low patient satisfaction, and increased adverse health events.^32^ A 2018 study in India found that language barriers contributed to greater workplace-related stress, delivery of low-quality services and low job satisfaction among healthcare professionals.^33^ A study in the United States highlighted that language barriers contribute to a poor understanding of health conditions, which sometimes results in misdiagnosis and delayed patients’ treatment.^34^

Rehabilitation therapists manage health conditions using the International Classification of Functioning, Disability and Health, which considers cultural practices under the personal factors component. However, many patients understood their health conditions from a spiritual rather than a personal perspective, as being punishments from supreme gods, which resulted in delays in initiating rehabilitation management, as they first sought assistance from traditional healers. The participants found these cultural views to be barriers to understanding the physiological causes of the disorders, which led to the refusal of trusted and tested treatment methods that would improve the disabled person’s quality of life. Ojok found that the cultural beliefs of the Yoruba-speaking patients in Nigeria regarded disabilities as being a supreme gift to live multiple realities both as humans and gods, and were therefore unwilling to consider rehabilitation service.^35^ A study conducted in Tanzania revealed that patients with disabilities live in fear for their lives because of cultural misconceptions by community members as the disabled person possesses supreme powers that can be harnessed.^36^

Participants also highlighted limitations with their profession-specific scopes of practice, particularly regarding medicine prescribing rights for conditions related to their profession, such as anti-inflammatory tablets, for which a prescription is not required. This limited the effectiveness of rehabilitation, as the scope to prescribe and provide medication during home visits would increase the effectiveness of their visits. Instead, patients had to visit a clinic to receive medication from the nursing sisters, who were allowed to provide limited medication, including anti-inflammatory tablets and antibiotics. Rehabilitation therapists in a number of countries are challenging the legislation that prevents them from prescribing and providing medication. Physiotherapists in Australia are currently expanding medicine prescribing rights through legislation and health models^37^ that will allow them to prescribe nonsteroidal anti-inflammatory analgesics and local anaesthetics medication. In 2022, Schwarz et al. in Australia investigated the feasibility of speech-language therapists prescribing specific categories of medicine and highlighted the pharmacological training needed to facilitate proper implementation.^38^ A South African study conducted in 2023 revealed that most physiotherapists support the inclusion of prescribing rights in their scope of practice and were aware of the required educational training.^39^

An important aspect of implementing social accountability in the workplace was the need to collaborate with community caregivers, who play an important role in assisting community members to maintain independence, dignity and quality of life. A local study recently reported that community caregivers in South Africa perform numerous cost-effective duties that help patients overcome some of the difficulties they experience when trying to access healthcare services, such as organizing rehabilitation service home visits and collecting medication on behalf of patients.^40^ A study conducted in the Mzinyathi District of KZN highlighted the need for sufficient support and funding for community caregivers as they assist with patient identification in their communities, and treatment access and monitoring.^41^ Participants in this study recommended collaboration with community caregivers to whom basic clinical skills could be transferred to aid in the early identification of people who would benefit from rehabilitation services, and help them to keep appointments.

Including rehabilitation services at primary health care facilities was also an important discussion in this study as part of being socially accountable. In the South African public health systems, primary health care clinics are the first point of contact, and commonly offer nursing care, nutrition and dietetics services, with referrals being required to access more specialised care, such as rehabilitation services. In rural areas, this means going to a district hospital, which may be some distance from home and require considerable effort to access, depending on the nature of the disability and the age of the affected person.^42^ Issues related to access can be a barrier to people seeking specialised care, and in the absence of mobile clinics that visit remote areas on a regular basis, affected families may not be able to afford the costs of transport, or have the capacity to get someone to a district hospital, specifically if they need to access this service on a regular basis. Including rehabilitation services at primary health care clinics would facilitate equitable service access and reduce the burden of patient care at referral institutions. While this does happen once a month, the time spent at each visit is insufficient to provide care to all those in need, with more visits being the solution to take services to where people need them as part of social accountability.

Participants felt that the lack of executive management support and the poor understanding of rehabilitation services from medical managers impacted the therapists’ ability to provide appropriate services. Generally, in community healthcare clinics and district hospitals, the medical managers are the executive heads of the rehabilitation departments and the therapists only link to more senior hospital executives, who decide on budgets and staffing allocations annually. As community service rehabilitation therapists are sometimes the only members of their department, their junior status and lack of experience make it difficult for them to advocate for departmental needs, which can result in their requirements not being regarded as priorities in resource-constrained settings. Studies have highlighted that the lack of management support has resulted in inadequate departmental funding, poor work monitoring and developmental feedback, overburdened rehabilitation therapists and a scarcity of senior rehabilitation therapists in many developing countries, which negatively impact service delivery at the district and community levels.^43,44^

As a result of the complexity of healthcare needs in communities, and the desire to be socially accountable, participants felt that CPD around issues relevant to community service therapists was essential. While some knowledge is discipline-specific, such as paediatric neurology, others are more related to transdisciplinary patient management, such as pharmacology, social services, logistics and neurodevelopment. Other authors have also emphasised the importance of continuing professional education for rehabilitation therapists in order to deliver ethical, high quality and evidence-based care in a rapidly evolving healthcare system.^45^ As many community service therapists worked alone and had no senior staff to advise them, the absence of formal instruction about the non-discipline topics that they would need to deal with should be addressed as part of their induction at the new institution or on an ongoing basis as part of CPD. As many people with disabilities can access government-funded disability grants, having information about how and where they can be accessed could be included in CPD courses. Issues such as how to accommodate differing conceptualisations of health and well-being could also be addressed in this way, thereby better enabling therapists to accommodate different belief systems when advising on the causes and treatment strategies available to increase daily functioning and increase quality of life.

### Limitations and strength

A number of limitations may have affected the findings, these being the small number of participants from each of the four disciplines, and that not all health districts in KZN were included. In addition, the study was restricted to rural and peri-urban areas, which means that the findings may not be applicable to urban areas. Despite these limitations, the use of FGDs and IDIs with participants across the province to enable data triangulation does strengthen the findings.

### Implications

Planning and implementation, budget and staff planning are essential components of being able to provide services to people where they need them, those with disabilities being particularly in need of being accommodated when annual budgets are determined to ensure that they are not disadvantaged by the system that is supposed to assist them.

Management issues and social accountability will not happen on their own and management issues need to be addressed to enable community service rehabilitation therapists to implement government guidelines. Curriculum development identifies areas that need to be addressed to prepare new graduates for the realities of working in rural and peri-urban public healthcare services.

## Conclusion

Working in a resource-constrained health setting can be a challenge for community-service rehabilitation therapists, particularly those who are on their own and have not been adequately prepared for the realities of rural and peri-urban service. Working in culturally diverse settings, with inadequate management support and scope of practice limitations can affect the type and quality of service they provide. As their patients are people with disabilities across the age spectrum, every effort needs to be made to provide the services they need, where and when they need them, given the potential to improve their quality of life and ability to live more independently. Understanding the rehabilitation therapist’s needs in their effort to provide socially accountable care is key to enabling them to reach those who require their services in communities that rely on public sector support.

### Recommendations

The recommendations of this study are as follows:

- Rehabilitation students at the undergraduate level need to be instructed about social accountability and how it affects the way they provide services within any community they work in, including those with diverse cultural conceptions of health.
- Universities need to include instruction in the provincially dominant languages and associated cultures in the health sciences curriculum to enable the various conceptualisations of health and healing to be accommodated when implementing social accountability of health services.
- More research is needed to identify how social accountability manifests in the public health sector, and how that can be accommodated during undergraduate theoretical and clinical training to prepare students for working in under-resourced contexts.
- Promoting inter-professional education and practice in higher education institutions would strengthen the public health systems where staff shortages would benefit from role release.
- Research is required into the types of medication the various rehabilitation disciplines need to dispense to motivate the HPCSA and South African Pharmacy Council (SAPC) for their inclusion as part of their scope of practice.
- Continuing professional education development modules need to be developed, in consultation with rehabilitation therapists, to provide information about the non-academic aspects that community service therapists need to appreciate in their public sector working environments.
- Given the additional difficulties that people with disabilities may have in accessing district-level services, greater effort needs to be made to provide more regular services closer to where they are needed.
